# Recent progress toward antiangiogenesis application of nanomedicine in cancer therapy

**DOI:** 10.4155/fsoa-2018-0051

**Published:** 2018-06-28

**Authors:** Sudip Mukherjee

**Affiliations:** 1 Department of Bioengineering, BioScience Research Collaborative, Rice University, Houston, TX 77005, USA

**Keywords:** angiogenesis, anti-angiogenesis, cancer therapy, nanomedicine, theranostics

Cancer is one of the foremost health problems worldwide, causing 8.2 million deaths and 14 million new cancer cases annually according to American Cancer Society statistics [1]. Moreover, as per the Forbes (Pharma & Healthcare) report, the market value for cancer is expected to increase up to approximately $147 billion by 2018 [2]. Conventional therapies for cancer involve radiation therapy, surgery, chemotherapy, photodynamic therapy, vaccinations, immunotherapy, stem cell transplantations or combinations of those. However, due to various limitations and side effects, development of an alternative therapeutic strategy is urgently required. In the recent past, nanomedicine has revolutionized the theranostics strategies for cancer due to their distinctive elemental properties (small size, high surface area, different interesting physicochemical properties and high surface energy) compared with bulk materials [3–5].

## Angiogenesis & its role in cancer

Angiogenesis is a complex physiological process that helps in the formation of new blood vessels from already existing vasculature [6]. It plays an important role in various pathological and physiological processes (embryonic development, tumor growth, organ growth and repair, wound healing, menstrual cycle and metastasis). The course of angiogenesis consists of different vital steps including endothelial cell proliferation, stimulation of endothelial cells by various endogenous growth factors, cell migration and capillary tube formation [7]. Disruption in the balance of angiogenesis can cause various disorders including malignancy, ischemic disease, immune disorders and infectious diseases. A healthy body always maintains overall balance of angiogenesis by maintaining the pro- and antiangiogenic signals. The processes by which drugs inhibit tumor growth by suppressing angiogenesis is called the antiangiogenesis procedure and is one of the cancer treatment strategies with the most potential.

Angiogenesis plays a vital role in tumor growth and progression, survival and metastasis. A primary tumor can grow only 1–2 mm^3^; beyond that it needs vascularization or angiogenesis to grow further. Moreover, tumor cells trigger an ‘angiogenic switch’ that attracts blood vessels from the nearby stroma, which is further controlled by several pro- or antiangiogeneic factors [8]. Several conditions can trigger the growth of a tumor including downregulation of angiogenic inhibitors, upregulations of angiogenic stimulators, hypoxic conditions and so on. Hence, blocking tumor angiogenesis could be an effective strategy for reduction of tumor growth. Conventional antiangiogenic therapy with various chemotherapeutic drugs (Avastin, Sunitinib, Imatinib, Pazopanib, Sorafenib, Axitinib, etc.) has several limitations including drug resistance, escaping VEGF-dependent angiogenesis, decreasing response for radiotherapy and so on. In this context, several nanoparticle-based antiangiogenic drug delivery systems have been developed by different research groups for the antiangiogenic cancer therapy including graphene oxides, gold nanoparticles, silver nanoparticles, carbon nanomaterials, cerium oxide nanomaterials, cuprous oxides nanoparticles, chitosan nanoparticles, lipid nanoparticles and more [9–11].

### Antiangiogenic nanomaterials for cancer therapy

Gold nanoparticles have long been used as cancer nanomedicine for their high biocompatibility, tunable size, easy synthesis, easy surface modifications, high drug loading and so on [12]. Mukherjee *et al*. showed the antiangiogenic property of 5 nm of spherical bare gold nanoparticles (AuNPs) for the first time [13]. The authors demonstrated that the AuNPs inhibit the activity of VEGF165 (HBGF) but do not interfere with the non heparin-binding VEGF121. Pan *et al*. recently observed that AuNPs inhibit the tumor angiogenesis by VEGF165-induced VEGFR2 and AKT phosphorylation. The authors also demonstrated the antitumor activity of AuNPs in mouse xenograft and ascites models [14].

Silica and silicate-based nanoparticles have also been used for antiangiogenic cancer therapy. Setyawati *et al*. recently showed the size-dependent antiangiogenic therapy of mesoporous silica nanoparticles that cause production of intracellular reactive oxygen species activating the p53 tumor suppressor pathway [15]. This resulting signaling cascade causes restriction in endothelial cell proliferation, migration and invasion and eventually impedes the tumor growth [15]. Song *et al*. reported the antiangiogenic properties of copper nanoparticles, which inhibit HUVEC cell proliferation, tube formation and cell migration, causing cell cycle arrest in a dose-dependent manner [16]. Moreover, the copper nanoparticles inhibited the VEGFR2 expression in a dose- and time-dependent manner that is verified in the protein and mRNA level.

Grodzik *et al*. demonstrated the antiangiogenic activities of ultradispersed detonation diamond nanoparticles in a glioblastoma multiforme tumor model developed on a chorioallantoic membrane [17]. The authors further showed that treatments with detonation diamond nanoparticles are associated with the downregulation of FGF and VEGFR, confirming the antiangiogenic activity. Mukherjee *et al*. recently demonstrated the dose-dependent modulation of angiogenesis or antiangiogenesis by the treatment of reduced graphene oxides and graphene oxides upon controlling the reactive oxygen species [18]. While the low doses of graphene oxides and reduced graphene oxides upregulated the phosphorylation of AKT and VEGF, the high doses downregulated these signaling cascades triggering the antiangiogenesis. This was confirmed by endothelial cell proliferation assay, tube formation assay, wound scratching assay and chicken embryonic angiogenesis assay.

Giri *et al*. showed that nanoceria (NCe) nanoparticles exhibit antiangiogenic activities upon inhibition of VEGF165-induced HUVEC proliferation, capillary tube formation, phosphorylation of VEGFR2 and activation of MMP2 [19]. The authors further demonstrated that NCe inhibited the growth of ovarian tumor in a preclinical mouse model, which was confirmed by vascular endothelial cell apoptosis and reduced CD31 staining. Hence, NCe could be successfully used for antiangiogenic agent in ovarian cancer therapy. In another published report, Gurunathan *et al*. observed the antiangiogenic properties of biosynthesized silver nanoparticles (AgNPs) [20]. These AgNPs showed antiangiogenic properties, inhibiting VEGF-induced endothelial cell proliferation, capillary-like tube formation and cell migration. Moreover, AgNPs inhibited the new blood microvessels in the mouse Matrigel plug assay through the activation of PI3K/AKT pathways. Apart from metallic and nonmetallic nanoparticles, several polymer-based nanoparticles were also successfully investigated for antiangiogenic applications in cancer therapy including chitosan nanoparticles, lipid-based nanoparticles, tetrac nanoparticles and peptide nanoparticles [11,21].

## Future perspective & challenges

The future challenge is to design and develop novel antiangiogenic nanomedicine that should exclusively target the cancer cells, without any side effects. Moreover, combination therapy using US FDA-approved chemotherapeutic drugs could help increase the anticancer efficacy of the antiangiogenic nanomaterials. Various issues need to be cautiously addressed before clinical implementation, including acute and chronic toxicity and pharmacokinetics and pharmacodynamics assessments of the nanomaterials, biodegradability and clearance of these nanomaterials, optimization of route of administration of dose, optimization of number of doses and frequency of doses, design of cost-effective and eco-friendly methods for the synthesis of antiangiogenic nanomaterials and strategies to combat drug resistance.

The distorted tumor vasculature offers adequate information for scientists to battle with to meet the challenges facing anticancer therapies. Special attention is necessary for effective antiangiogenic therapies in terms of metastasis, invasiveness and overall survival. With a rising number of antiangiogenic cancer agents being considered for clinical approval or approved, the call for biomarkers is vital for measuring safety and efficacy. The recent progress of nanobiotechnology offers paramount opportunities for researchers for the development of new-age technology to combat cancer. However, the future of antiangiogenic cancer treatment using nanomedicine needs additional knowledge regarding physiological barriers, cancer cell metabolic properties and other important information related to materials properties in order to improve its anticancer efficiency.

## References

[1] Global Cancer Facts & Figures (2018). *American Cancer Society*.

[2] Herper M (2015). *Forbes, Pharma & Healthcare*.

[3] Ferrari M (2005). Cancer nanotechnology: opportunities and challenges. *Nat. Rev. Cancer*.

[4] Mukherjee S, Das S, Nuthi S, Patra CR (2017). Biocompatible nickel-prussian blue@ silver nanocomposites show potent antibacterial activities. *Future Sci. OA*.

[5] Mukherjee S, Patra CR (2017). Biologically synthesized metal nanoparticles: recent advancement and future perspectives in cancer theranostics. *Future Sci. OA*.

[6] Carmeliet P (2005). Angiogenesis in life, disease and medicine. *Nature*.

[7] Yancopoulos GD, Davis S, Gale NW (2000). Vascular-specific growth factors and blood vessel formation. *Nature*.

[8] Caporarello N, Lupo G, Olivieri M (2017). Classical VEGF, notch and Ang signalling in cancer angiogenesis, alternative approaches and future directions. *Mol. Med. Rep.*.

[9] Mukherjee S, Patra CR (2016). Therapeutic application of antiangiogenic nanomaterials in cancers. *Nanoscale*.

[10] Hashemi GN, Ghiyami-Hour F, Jahangiri S (2018). Nanoparticles as new tools for inhibition of cancer angiogenesis. *J. Cell Physiol.*.

[11] Abdalla AM, Xiao L, Ullah MW, Yu M, Ouyang C, Yang G (2018). Current challenges of cancer antiangiogenic therapy and the promise of nanotherapeutics. *Theranostics*.

[12] Mukherjee S, Nethi SK, Patra CR (2017). Green synthesized gold nanoparticles for future biomedical applications. *Particulate Technology for Delivery of Therapeutics*.

[13] Mukherjee P, Bhattacharya R, Wang P (2005). Antiangiogenic properties of gold nanoparticles. *Clin. Cancer Res.*.

[14] Pan P, Ding H, Qin L (2013). Gold nanoparticles induce nanostructural reorganization of VEGFR2 to repress angiogenesis. *J. Biomed. Nanotechnol.*.

[15] Setyawati MI, Leong DT (2017). Mesoporous silica nanoparticles as an antitumoral-angiogenesis strategy. *ACS Appl. Mater. Interfaces*.

[16] Song H, Wang W, Zhao P (2014). Cuprous oxide nanoparticles inhibit angiogenesis via downregulation of VEGFR2 expression. *Nanoscale*.

[17] Grodzik M, Sawosz E, Wierzbicki M (2011). Nanoparticles of carbon allotropes inhibit glioblastoma multiforme angiogenesis in ovo. *Int. J. Nanomed.*.

[18] Mukherjee S, Sriram P, Barui AK (2015). Graphene oxides show angiogenic properties. *Adv. Healthc. Mater.*.

[19] Giri S, Karakoti A, Graham RP (2013). Nanoceria: a rare-earth nanoparticle as a novel anti-angiogenic therapeutic agent in ovarian cancer. *PLoS ONE*.

[20] Gurunathan S, Lee KJ, Kalishwaralal K (2009). Antiangiogenic properties of silver nanoparticles. *Biomaterials*.

[21] Gu G, Hu Q, Feng X (2014). *Biomaterials*.

